# Prevalence of Covert Hepatic Encephalopathy in A Tertiary Care Centre

**DOI:** 10.31729/jnma.4809

**Published:** 2020-01-31

**Authors:** Rahul Pathak, Pukar Ghimire, Sabin Thapaliya, Sashi Sharma, Prem Khadga

**Affiliations:** 1 Department of Gastroenterology, Institute of Medicine, Maharajgunj, Nepal; 2 Department of Internal Medicine, College of Medical Sciences, Chitwan, Nepal; 3 Department of Internal Medicine, Institute of Medicine, Maharajgunj, Nepal

**Keywords:** *chronic liver disease*, *covert hepatic encephalopathy*, *minimal hepatic encephalopathy*, *number connection test*, *overt hepatic encephalopathy*

## Abstract

**Introduction:**

Among patients with Hepatic Encephalopathy, prevalence of Minimal HE varies between 30-50%. Identifying patients with MHE has been shown to improve with medications and delay development of Overt HE, however only limited clinicians screen for MHE in patients due to time consuming neuropsychological and neurophysiological tests. The Number Connection Test is an easy way to evaluate patients to diagnose MHE. The aim of this study is to find out the prevalence of covert hepatic encephalopathy.

**Methods:**

The descriptive cross-sectional study was done to find out the prevalence of covert hepatic encephalopathy among patients with chronic liver disease. To diagnose Covert HE which included MHE as well, NCT was used in Devanagari script.

**Results:**

The prevalence of covert hepatic encephalopathy is found to be 56 (58.3%) at 90% confidence interval (58.23-58.37%). A total of 96 patients (71.9% male) were diagnosed as HE, with mean age of 49.6+11.8 years. The cause of CLD in 85 (88.5%) of these patients was alcohol, of which 76 (79.2%) consumed locally brewed alcohol. Of these 96 patients with HE, only 40 (41.7%) had overt HE. Among all these, maximum patients had MHE (37.5%).

**Conclusions:**

Our study showed that although the prevalence of minimal HE is quite high among cirrhotics, they are usually missed in clinical practice due to absence of symptoms. Active screening with easy-to-administer tests, like Number Connection tests, can help identify patients with minimal HE and hence treat them early.

## INTRODUCTION

HE (Hepatic Encephalopathy) is associated with poor survival and a high risk of recurrence.^1,2^ HE reduces quality of life and predisposes to severe HE.^3^ Overt HE (OHE) is found in 10-14% cirrhotic at diagnosis.^4,5^ MHE is found in 30-50% cirrhotic depending on the diagnostic criteria.^6,7^ Only limited clinicians screen patients for MHE for various reasons.^2,8^

Correlation between severity of liver disease and prevalence of MHE remains controversial.^9,10^ MHE and CHE (Covert HE) are the presence of test-dependent or clinical signs of brain dysfunction without disorientation or asterixis. Covert HE includes minimal and grade 1 HE.^11^ Testing strategies can be psychometric and neurophysiological.^12^ Patients with subtle problems are advised to be tested.^13^ Porto-systemic encephalopathy (PSE) syndrome tests are commonly used because of easy administration and good external validity.^14^ Although not commonly used by clinicians, Number connection test(NCT) A and B are easy to administer and help diagnose MHE.^15^

The main aim of this study is to find out the prevalence of covert hepatic encephalopathy among patients with chronic liver disease in a tertiary care centre of Nepal.

## METHODS

The descriptive cross-sectional study was carried out in Tribhuvan University Teaching Hospital from June 2017 to June 2018. Ethical clearance from IRB (Institutional Review Board) for the study was taken.

The sample size was calculated as follows:

$$\begin{array}{ll} \text{Sample size }(\text{n)} & \text{= }{\text{Z}}^{\text{2}}\times \text{pq}/{\text{e}}^{2}\\ & \text{= }{(\text{1.64)}}^{\text{2}}\times \text{0.5}\times \text{(1}-\text{0.5)}/{(\text{0.1)}}^{\text{2}}\\ & \text{= }67 \end{array}$$

Where,

Confidence Interval (CI) = 90%
Margin of error (e) = 10%
p = prevalence which was taken as 50%
q = (1-p)

Therefore, the calculated sample size was 67. Adding the 10% non-response rate, the sample size that will be taken would be 74. Convenience sampling method has been applied.

All patients aged > 18 with chronic liver disease were included.

The exclusion criteria were CKD (Chronic Kidney disease) with creatinine >2mg/dl, sedatives within last 7 days, medical or psychiatric conditions interfering with assessments and those who did not give consent. A total of 96 patients with chronic liver disease who fulfilled the inclusion criteria were taken.

We designed this study to diagnose HE among patients with chronic liver disease and find the proportion of grades of HE as per West Haven criteria. To diagnose covert HE, Number connection test was used in Devanagari script.

All patients with Chronic liver disease were evaluated for hepatic encephalopathy. Patients exhibiting gross features like flaps and disorientation were classified as Overt encephalopathy, those patients without these features were subjected to NCT. A patient completing the NCT with more than 30 seconds were classified as Minimal Hepatic encephalopathy. After analyzing the sleep pattern or cognitive and behavioral disturbances Grade I hepatic encephalopathy was classified.

Data collected was kept in Microsoft Excel and then edited and checked. After that the data was put in SPSS. Frequency, percentages was calculated for binary data and mean and standard deviation was calculated for continuous data after the normality of the data has been checked.

## RESULTS

The prevalence of covert hepatic encephalopathy is found to be 56 (58.3%) at 90% confidence interval (58.23-58.37%). The baseline characteristics of the patients diagnosed as HE were as follows (Table 1). Of 96 patients with HE, only 40 (41.7%) had overt HE, the remaining 56 (58.3%) had covert HE.

**Table 1:** Baseline Characteristics of the patients diagnosed as HE.

Characteristics	n (%)
Sex	
Male (%)	69 (71.9)
Female (%)	27 (28.1)
Age in years (Mean±SD)	49.6±11.8

The most common cause of CLD was found to be alcohol 85 (88.5%) (Table 2) and the most often consumed alcohol is locally brewed 76 (79.2%) followed by factory branded 9 (9.4%).

**Table 2:** Causes of Chronic Liver Disease.

S.N	Causes of CLD	n (%)
1.	Alcohol	85 (88.5)
2.	Viral Hepatitis	8 (8.3%)
3.	Others	3 (3.2%)

**Figure 1.**
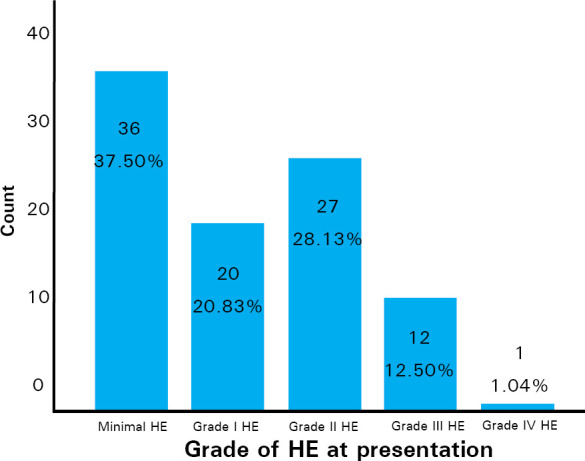
Bar diagram showing grades of HE at presentation.

Around 36 (37.5%) patients were in minimal hepatic encephalopathy stage and the next most patients were in grade II hepatic encephalopathy (Figure 1).

Among the precipitants of hepatic encephalopathy, gastrointestinal bleeding 31 (32.3%) and others 30 (31.2%) were the major one followed by infection, electrolyte disorder and constipation.

## DISCUSSION

Chronic liver disease is a common problem. Alcohol is the most common cause of CLD in our study and this represents the scenario of the whole population. Almost 80% of pateints in this study were found to be consuming locally brewed alcohol, also known as ‘Local’ Alcohol. A study done in Nepal found that two types of homebrewed alcohol are readily available, namely “Distilled” (local Raksi) and “Non-distilled” (Jand, Chhyang, Tumba). Concentration of ethanol in those preparations ranged from 3% to 40% for distilled, and 1% to 18.9% for non-distilled.^16^ Other group of investigators in Nepal, reported alcohol accounting for 60.8% of CLD.^17^

Of several complications of chronic liver disease, hepatic Encephalopathy is an important one. 14.9% of patients with CLD are reported to have HE in previous study in Nepalese population.^17^

Overt HE is associated with significant clinical features so the treatment results are obvious. In spite of being grossly asymptomatic, covert HE also has an implication.

There is compelling evidence in the literature to suggest that MHE has a profound impact on patients daily functioning and well-being. About half of the patients with MHE may be unfit to work and may not have regular employment.^18^

We had maximum number of patients in MHE (37.5%), followed by grade II group HE (28.1%) and then by HE Grade I (20.8%). Previous studies have also similar findings, except that MHE were not into sight. The large variation in previously reported prevalence of MHE (22-84 %) is because of different diagnostic criteria used and the patient population studied.^8,13,19^

By using Number connection test, we could diagnose 37.5% patients as having MHE, which would not have been possible. Treating those patients and reversing HE, impacts the quality of their life. However, the effect of that impact needs further study for quantification.

Further, this study was the first to use number connection test in Devanagari script to diagnose covert HE. We could show that we can modify the number connection test in our local script so that it can be used in any communities we are practicing in.

Limitations of the study were also evaluated. We appreciated the fact that, although our patients can read numbers, they are not used to using pen and paper, so it is possible that some of the cases with minimal HE might have been exaggerated. The sample size is less which could bring better results in larger sample size.

## CONCLUSIONS

Our study showed that although the prevalence of minimal HE is quite high (30-50%) among cirrhotics, they are usually missed in clinical practice due to absence of symptoms. Active screening with easy-to- administer tests, like Number Connection tests, can help identify patients with minimal HE and hence treat them early. NCT can be modified in Local script and hence, can be used in any part of the world.

## Conflict of Interest

**None.**
